# BMI1, Stem Cell Factor Acting as Novel Serum-biomarker for Caucasian and African-American Prostate Cancer

**DOI:** 10.1371/journal.pone.0052993

**Published:** 2013-01-07

**Authors:** Hifzur Rahman Siddique, Aijaz Parray, Weixiong Zhong, R. Jeffery Karnes, Eric J. Bergstralh, Shahriar Koochekpour, Johng S. Rhim, Badrinath R. Konety, Mohammad Saleem

**Affiliations:** 1 Molecular Chemoprevention and Therapeutics, The Hormel Institute, University of Minnesota, Austin, Minnesota, United States of America; 2 School of Medicine and Public Health, University of Wisconsin, Madison, Wisconsin, United States of America; 3 Department of Urology, Mayo Medical School and Mayo Clinic, Rochester, Minnesota, United States of America; 4 Division of Biomedical Statistics and Informatics, Mayo Medical School and Mayo Clinic, Rochester, Minnesota, United States of America; 5 Center for Genetics and Pharmacology, Roswell Park Cancer Institute, Buffalo, New York, United States of America; 6 Department of Surgery, Uniformed Services University of the Health Sciences, Bethesda, Maryland, United States of America; 7 Department of Urology, University of Minnesota, Minneapolis, Minnesota, United States of America; 8 Department of Laboratory Medicine Pathology, University of Minnesota, Minneapolis, Minnesota, United States of America; University of Kentucky College of Medicine, United States of America

## Abstract

**Background:**

Lack of reliable predictive biomarkers is a stumbling block in the management of prostate cancer (CaP). Prostate-specific antigen (PSA) widely used in clinics has several caveats as a CaP biomarker. African-American CaP patients have poor prognosis than Caucasians, and notably the serum-PSA does not perform well in this group. Further, some men with low serum-PSA remain unnoticed for CaP until they develop disease. Thus, there is a need to identify a reliable diagnostic and predictive biomarker of CaP. Here, we show that BMI1 stem-cell protein is secretory and could be explored for biomarker use in CaP patients.

**Methodology/Principal Findings:**

Semi-quantitative analysis of BMI1 was performed in prostatic tissues of TRAMP (autochthonous transgenic mouse model), human CaP patients, and in cell-based models representing normal and different CaP phenotypes in African-American and Caucasian men, by employing immunohistochemistry, immunoblotting and Slot-blotting. Quantitative analysis of BMI1 and PSA were performed in blood and culture-media of siRNA-transfected and non-transfected cells by employing ELISA. BMI1 protein is (i) secreted by CaP cells, (ii) increased in the apical region of epithelial cells and stromal region in prostatic tumors, and (iii) detected in human blood. BMI1 is detectable in blood of CaP patients in an order of increasing tumor stage, exhibit a positive correlation with serum-PSA and importantly is detectable in patients which exhibit low serum-PSA. The clinical significance of BMI1 as a biomarker could be ascertained from observation that CaP cells secrete this protein in higher levels than cells representative of benign prostatic hyperplasia (BPH).

**Conclusions/Significance:**

BMI1 could be developed as a dual bio-marker (serum and biopsy) for the diagnosis and prognosis of CaP in Caucasian and African-American men. Though compelling these data warrant further investigation in a cohort of African-American patients.

## Introduction

According to American Cancer Society, 241,740 will be diagnosed with prostate cancer (CaP) and 28,170 CaP patients were projected to die in the year 2012 in USA alone [1]. The unsatisfactory outcome of overall management (treatment strategies and prognosis monitoring) for CaP disease could be associated to the lack of a reliable prognostic serum-biomarker. Although widely used, several important caveats have been reported in serum-PSA as a prognostic biomarker [2]. For example, in some CaP cases, serum-PSA is (a) detected little if any, (b) lacks adequate sensitivity, and (c) fails to discriminate potentially significant cancers from insignificant ones [2]–[4]. PSA does not reflect cancer biology and a high risk of mistaken results [5]–[6]. Further, discrepancies in PSA as a diagnostic marker among different racial groups such as Caucasians and African-American have confounded the management of this cancer [6]–[7]. Therefore a great need persists for the development of improved serologic biomarkers in CaP, which is reliable for prognosis and diagnosis in Caucasian and African-American patients.

There is increasing evidence that polycomb group (PcG) proteins play a crucial role in cancer development and disease recurrence [8]. B-cell-specific Moloney murine leukemia virus integration site 1 (BMI1) is a well-known marker used in stem cell biology [8]–[9]. BMI1 which has an ubiquitous pattern of expression in almost all tissues is frequently upregulated in various types of human cancers [8]–[10]. We recently reviewed significance of BMI1 in the emergence of chemoresistance in various types of cancers including CaP [8]. The current study is the first clinical evidence showing that BMI1 is a secretory protein that has tremendous potential to be developed as a serum-biomarker for CaP and its prognosis in both Caucasian and African-American population. We suggest that serum-BMI1 as a biomarker would perform better than PSA. Further, BMI1 could be used as a dual biomarker in serum as well as biopsy.

## Materials and Methods

### Prostate tissues and Serum samples from human CaP patients

Prostatic tissues surgically harvested from human CaP patients and matching paraffin blocks were procured from Cooperative Human Tissue Network Midwestern Division, The Ohio State University (Columbus, OH). Serum samples of human CaP patients were procured from serum bank (BioServe, Beltsville, MD). Additional paraffin-embedded sections of human prostate tissues of 70 patients with normal and adenocarcinoma were obtained from the ISU Abxis Co. Ltd., (Seoul, South Korea).

### Cell Lines

Cell lines originated from both Caucasian and African American mans were used in our study. Normal and immortalized prostate epithelial cell line (RWPE1), CaP cell lines (LNCaP, C42b, PC3, Du145, VCaP and PCa-2b), prostatic stromal myofibroblasts (WPMY1), colon normal epithelial cells (FHC) and colon cancer cell lines (SW480, HCT116 and HT29) and human pancreatic carcinoma cell lines PANC1 and AsPC1 were obtained from ATCC (Manassas, VA). Normal pancreatic ductal epithelia cells, premalignant Kras mutant E6E7-Ras and malignant Kras mutant E6E7-Kras-st cells were obtained from D. Paul M. Campbell (H. Lee Moffitt Cancer Center, Tampa, FL) [11]. BPH-1 cells were procured from Dr. Simon Hayward (Vanderbilt University, Nashville, TN) who developed them as described [12]. Establishment and characterization of RC77N/E, RC77T/E and E006 cells was described earlier [13]–[14]. Cells were grown in appropriate media supplemented with 10% FBS (ATCC, Manassas, VA) and 1% Penicillin-Streptomycin (Invitrogen, Carlsbad, CA) under standard cell culture conditions of 5% CO_2_ in an incubator at 37°C.

### Cell Selection

(a) Caucasian Cells: RWPE1 (normal), BPH-1 (non-malignant hyperplasia) and, LNCaP, C4-2B, PC-3, Du145 and VCaP representing Caucasian prostate cancer. WPMYI1 stromal fibroblasts were also used. (b) African American Cells: RC77N/E (normal), and RC77T/E, PCa-2B, E006 representing African American prostate cancer.

### Antibody, Plasmids and siRNA

Monoclonal anti-BMI1 antibody was procured from Millipore (Temecula, CA). pbabe-BMI1 plasmid (BMI1-overexpressing) was a kind gift from Dr. Chi V. Dang (The John Hopkins University, Baltimore, MD). BMI1-siRNAs were commercially purchased from Dharmacon (Lafayette, CO).

### Immunohistochemistry

Immunohistochemical staining was performed as described earlier [15]–[16]. Briefly, paraffin sections (to be evaluated for BMI1) were pretreated with citrate buffer (pH 6) for 10 min in a microwave for antigen retrieval. Sections were incubated with primary antibody (anti-BMI1) at a dilution of 1∶50 for 12 h at 4°C. Slides were then incubated for 2 h at room temperature with appropriate HRP-conjugated secondary antibody. Slides were developed in 3, 3′-diaminobenzidene (DAB kit, Invitrogen, Carlsbad, CA) and counter stained with hematoxylin. The stained slides were dehydrated and mounted in permount solution under cover slips.

### Western blot Analysis

Immunoblots analysis was performed as described earlier [16]–[17]. Briefly, cell lysates were prepared in cold lysis buffer [(0.05 mmol/L Tris-HCl, 0.15 mmol/L NaCl, 1 mole/L EGTA, 1 mol/L EDTA, 20 mmol/L NaF, 100 mmol/L Na_3_VO4, 0.5% NP-40, 1% Triton X-100, 1 mol/L phenyl methylsulfonyl flouride (pH 7.4)] with protease Inhibitor Cocktail (Roche, Indianapolis, IN). The lysate was collected and stored at −80°C. The protein content in the lysates was measured by BCA protein assay (Pierce, Rockford, IL), as per the vendor's protocol. For Western blot analysis, 40 µg protein was resolved in 10% SDS-PAGE gels, transferred onto PVDF membranes (Millipore, Bedford, MA) and subsequently incubated in blocking buffer (5% nonfat dry milk/1% Tween 20; in 20 mmol/L TBS, pH 7.6) for 2 hours. The blots were incubated with BMI1 primary antibody, washed and incubated with HRP-conjugated secondary antibody (Sigma, Saint Louise, MO). The blots were detected with chemiluminescence (ECL kit, Amersham Biosciences, Piscataway, NJ). Equal loading of protein was confirmed by stripping the blots and re-probing with β-actin (Sigma, St. Louis, MO). Densitometry measurements of the scanned bands were performed as described earlier [16].

### Detection of Protein in cell culture media

Cells were allowed to grow up to 80% confluence in complete media. At 80% confluent level, media was discarded and cells were washed with PBS twice. After washing, cells were added with serum-free media. Cells were cultured in serum-free media for 24 h. After 24 h, media was collected and analyzed for BMI1 secretory protein by using Immuno-Slot-blot assay. The Slot-blot assay was performed as per the manufacturers' protocol (Whatman, Florham Park, NJ). Briefly, Slot-blot apparatus was assembled using Whatman filter paper and a pre-wetted nitrocellulose membrane. Next, the apparatus was connected to a vacuum pump. Slots were filled with samples (media/serum) and then drawn by vacuum (unused slots were filled with PBS). The membranes were then blocked for 2 h in blocking buffer (5% nonfat dry milk). The blots were incubated with BMI1 primary antibody, washed and incubated with HRP-conjugated secondary antibody (Sigma, Saint Louise, MO). The blots were detected with chemiluminescence (ECL kit, Amersham Biosciences).

### Removal of Albumin from serum samples

Albumin was removed from human serum samples by using Albumin Removal Kit (Pierce, Rockford, IL) as per vendor's protocol. Samples containing 1000 µg of total protein were loaded onto a single removal disc, where each disc is reported to have a binding capacity of >2 mg of albumin.

### Estimation of PSA protein levels by ELISA

This was performed by using human PSA-specific ELISA (Anogen, Ontario, Canada) as per vendor's protocol.

### Quantification of secretory BMI1 protein in culture media

This was performed by using a BMI1-specific ELISA (Antibodies-online Inc., Atlanta, GA). Recombinant BMI1 protein was used to serve as standard for this assay.

### BMI1-siRNA and pbabe-BMI1 (BMI1-expressing plasmid) transfection to validate that BMI1 is indeed secreted by CaP cells

Transfections were performed by using Lipofectamine (Invitrogen, Carlsbad, CA) as per vendor's protocol. For this reason, first intracellular BMI1 from Caucasian CaP (LNCaP and Du145) and African American CaP (E006) epithelial cells was determined.

#### Under 1^st^ approach

BMI1 was knocked down by shRNA in Caucasian and African American cells. 12 h after transfection, cells were grown in complete media for 12 h. After 24 h post-transfection, media was discarded and cells were grown in serum-free media for 24 h. After 24 h, serum-free media from BMI1-knockdown cells was collected and secreted-BMI1 levels were measured by ELISA.

#### Under 2^nd^ approach

prostate cancer cells representing Caucasian and African American disease were transfected with BMI1-overexpressing plasmid. 12 h after transfection, cells were grown in complete media for 12 h. After 24 h post-transfection, media was discarded and cells were grown in serum-free media for 24 h. After 24 h, serum-free media from BMI1-overexpressing cells was collected and secreted-BMI1 levels were measured by ELISA.

### Androgen treatment of cells

For this reason, Caucasian and African American prostate epithelial cells were treated with androgen analogue (R1881) for 12 h. After 12 h, media was discarded and cells were grown in further 12 h. After 24 h, cells were harvested to be evaluated for intracellular BMI1 expression by western blot analysis.

### Statistical analyses

Graphical summaries of the distribution of staining intensity were made using scatter plots and box plots. Simple linear egression and correlation methods were use to evaluate associations between BMI1, PSA and CaP rank (1 = normal, 2 = Stage II, 3 = Stage III, 4 = Stage IV). To correct for skewness, BMI1 and PSA were analyzed on a log (base2) scale. A p-value of <0.05 was considered to be statistically significant.

## Results

### Bmi1 protein levels in prostatic tissues increases with progressive stages of disease in transgenic TRAMP mouse models

**Figure 1:**
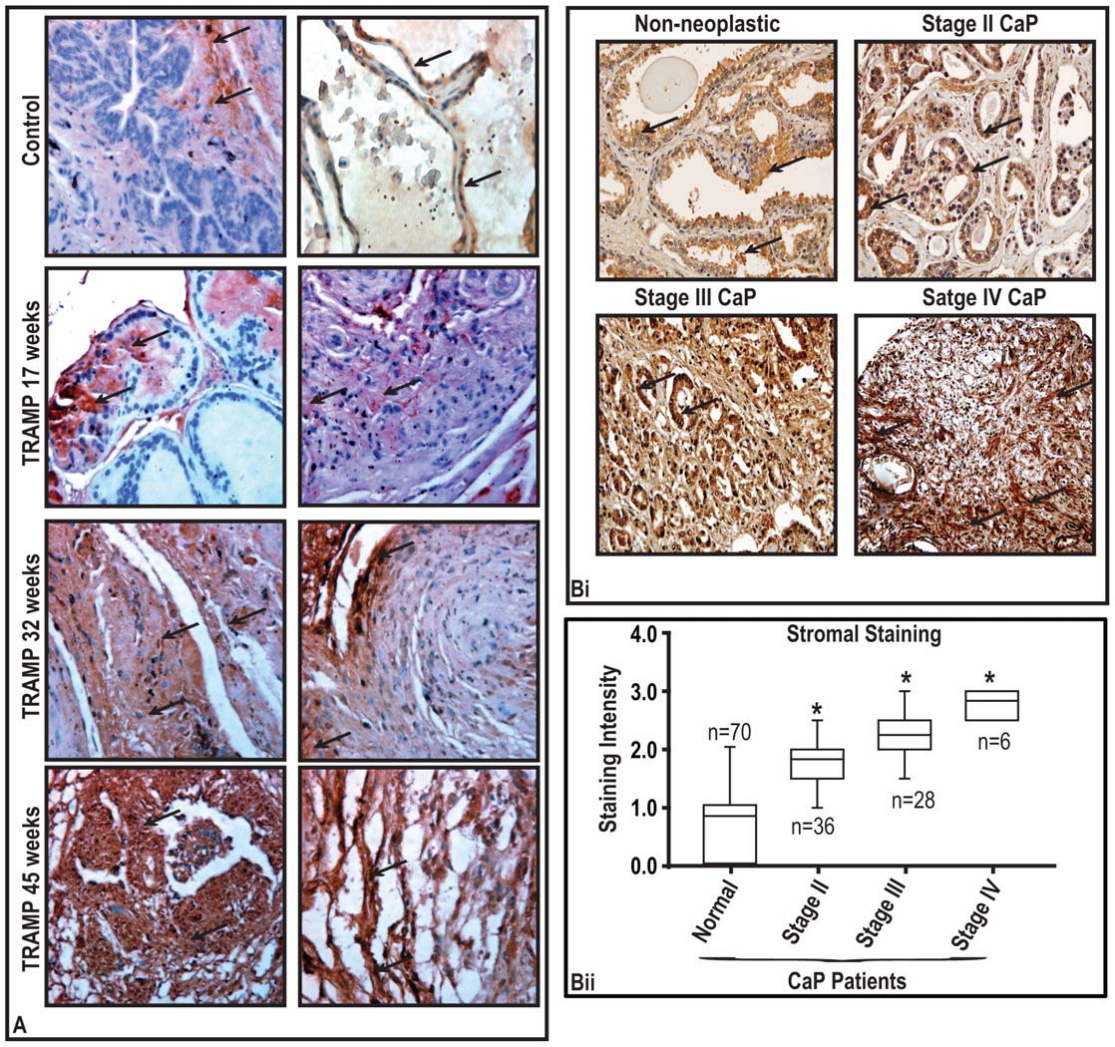
BMI1 protein levels in prostatic tumor tissues of humans and TRAMP transgenic mice. (**A**) Photomicrographs represent immunostaining of BMI1 in prostatic tissues of transgenic TRAMP mice. Arrows indicate staining for BMI1. Magnification ×40. (**Bi**) Photomicrographs show BMI1-positive neoplastic and non-neoplastic regions of prostatic specimens of CaP patients as assessed by immunostaining. Arrows indicate staining for BMI1. Magnification ×40. (**Bii**) Box plots for BMI1 protein based on score pertain to immunostaining pattern in normal and CaP specimens in stromal region.*, P<0.05; black bar in box, median values.

Glinsky et al. [18] previously showed that Bmi1 protein is elevated in the prostatic tissues of TRAMP mice, an autochthonous mouse model of CaP development, we investigated if a progressive increase in the levels of Bmi1 in prostatic tissues could be detected during progressive age of CaP. For this purpose we used prostatic tissue samples collected at different ages of TRAMP transgenic mice. As shown in Fig 1A; Bmi1 protein was observed to be detectable in all ages of TRAMP mice. In general, the staining was stronger in prostatic epithelial cells from older mice than in prostatic epithelial cells from younger mice. Smooth muscle cells have much stronger staining than fibroblast cells. The staining pattern of Bmi1 protein was compared in age 17 weeks to 45 weeks old prostatic specimens (Fig. 1A). These data showed increased expression levels of Bmi1 protein in prostate of older aged mice (Fig. 1A). There was an intense staining at apical region of epithelial cells. Stromal regions were observed to have a positive staining (Fig. 1A).

### BMI1 protein expression in prostatic tissue specimens of CaP patients

Notably, some epithelial cells of transgenic mouse prostate epithelial cells showed dense apical staining suggesting that Bmi1 could be a secretory protein. We next identified the expression of BMI1 in human CaP specimens by immunohistochemical analysis and determined its expression levels in stromal regions of 70 pair-matched specimens of normal and CaP representing all tumor stages. The intensity of immunoperoxidase staining for BMI1 was scored as 0 (negative), 1 (weak), 2 (moderate) and 3 (strong). Immunostains showed staining in both non-neoplastic and neoplastic stroma. In general, the staining was stronger in neoplastic stroma than in non-neoplastic stroma. The epithelial cells also showed positive staining for the antibody (Fig. 1Bi). Smooth muscle cells have much stronger staining (3) than fibroblast cells (0–1+). The staining pattern of BMI1 protein was compared in stage II–IV CaP specimens (Fig. 1Bi). These data showed increased expression levels of BMI1 protein in high grade tumor in human CaP (Fig. 1Bi). The box plots of the data for BMI1 protein expression in stroma exhibited a wide inter-specimen variation in cancer specimens, compared with normal tissues and revealed a significant difference in the level of protein between normal and CaP tissues (p<0.05, Fig. 1Bii). The average score for the staining intensity of BMI1 in stroma of normal tissues was 0.81±0.07 (n = 70), and was significantly lower than high-grade stage II (1.8±0.08; n = 36), stage III (2.26±0.10 n = 28) and stage IV (2.8±0.11; n = 6) cancer specimens (Fig. 1Bii; p<0.05). A similar pattern of staining in pair-matched CaP specimens was observed in the epithelial of the prostatic specimens. Taken together, these data show that expression of BMI1 increases with increasing stage of CaP.

### BMI1 expression in normal and neoplastic prostatic cells representing CaP disease in Caucasian men

**Figure 2:**
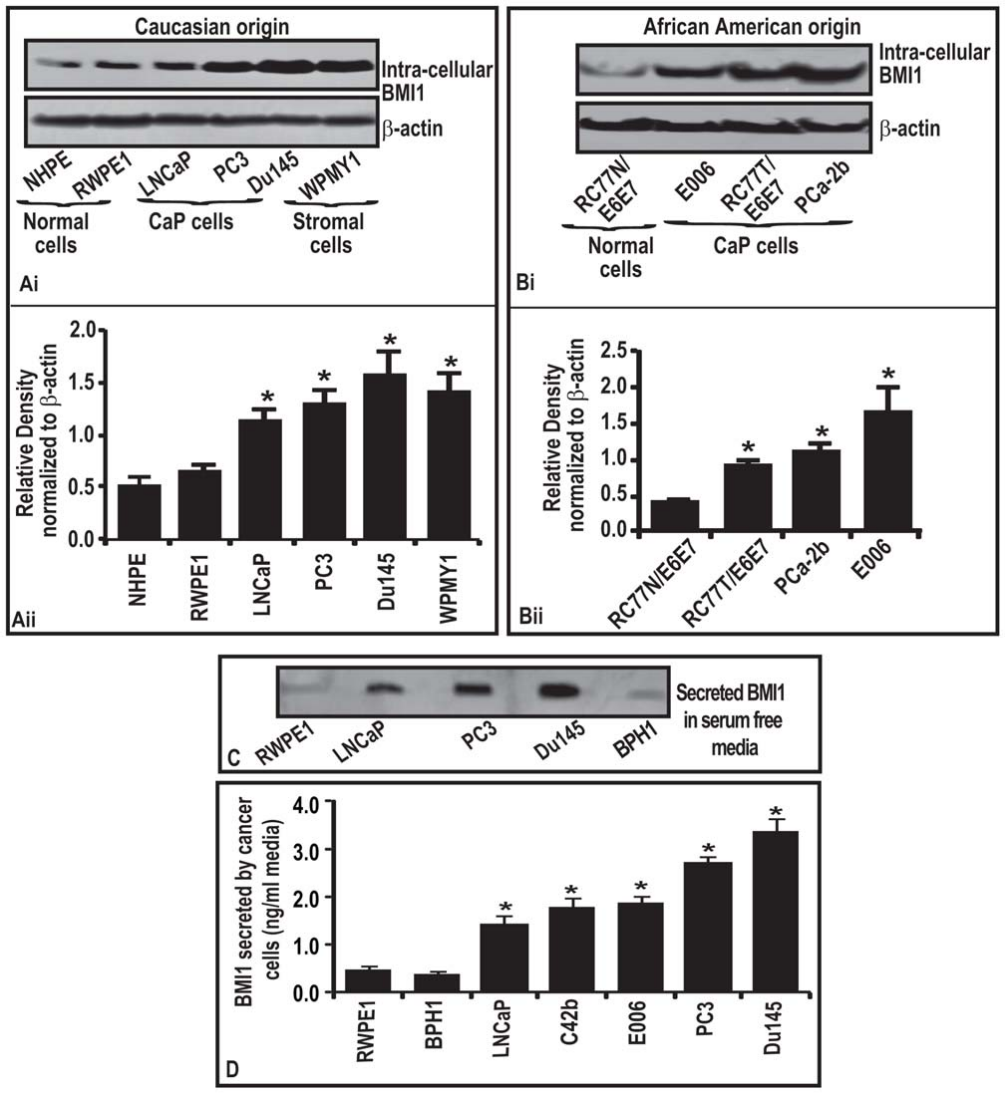
BMI1 protein levels (in both intracellular and secretory forms) correlate to the aggressiveness of tumor cell type representing Caucasian and African American CaP disease. (**Ai**) Figure represents the level of BMI1 protein in normal and CaP cells of Caucasian origin as assessed by immunoblot analysis. Equal loading of protein was confirmed by reprobing immunoblot for β-actin. The blot shown here are representative of three samples. (**Aii**) Histogram showing the densitometry analysis of immunoblots of BMI1. *, P<0.05; black bar in gray box, median values. (**Bi**) Figure represents the level of BMI1 protein in normal and CaP cells of African American origin as assessed by immunoblot analysis. Equal loading of protein was confirmed by reprobing immunoblot for β-actin. The blots shown here are representative of three samples. (**Bii**) Histogram showing the densitometry analysis of immunoblots of BMI1. *, P<0.05; black bar in gray box, median values. (**C**) Figure represents the detection of BMI1 in conditional culture medium of different cells as assessed by Slot-blot analysis. The blots data shown here are representative of three samples. (**D**) Detection of secreted BMI1 protein in conditioned culture medium of cells. Each bar in the histogram represents mean ± SE of 3 independent experiments, *represents P<0.05.

As an attempt towards identifying the expression of BMI1 in CaP progression, we first measured protein expression levels by immunoblot analysis in several human Caucasian CaP cell lines, LNCaP, Du145 and PC3 and compared them to NHPE (normal primary prostate epithelial cell) and RWPE1 (representing normal immortalized prostatic epithelial cells), respectively. Among the CaP cell lines used, LNCaP is androgen-dependent whereas Du145 and PC3 are androgen-independent. The choice of these cells was based on the fact that 80% CaP patients present with androgen-dependent disease at the time of diagnosis which later transforms into more aggressive, androgen-independent disease [19]. As shown in Figure 2(Ai–ii), all CaP cell lines exhibited a higher expression of BMI1 protein than in normal prostate epithelial cells. When the protein expression of BMI1 was compared, based on the densitometric analysis of the immunoblots, highly aggressive PC3 cells and Du145 exhibited higher expression than in LNCaP cells (Fig. 2Aii). Interestingly, we also detected BMI1 expression in the prostate stromal cells (WPMY1) (Fig. 2Ai–ii). Interestingly, BMI1 expression was found to be very low in prostate epithelial cells representing benign prostatic hyperplasia (BPH) condition (data not shown).

### BMI1 expression in normal and neoplastic prostatic cells representing CaP disease in Africa- American men

Age-adjusted data from SEER study showed that African-American men have a 60% higher incidence and 125% higher mortality rates from CaP than Caucasian men [1], [13]. Race and family history are the two most widely accepted risk factors for this disease [1]. Understanding the underlying biological mechanisms responsible for CaP progression will eventually lead to the development of more effective therapeutic strategies. We determined the levels of BMI1 expression in a cell-based in vitro model representing different phenotypes of CaP disease in African-American men. These include RC77N/E (representing normal prostatic epithelial cells in African-American men), RC77T/E (representing androgen-dependent tumorigenic prostatic epithelial cells), E006 (representing androgen-dependent non-tumorigenic prostatic epithelial cells) and PCa-2b (representing CRPC phenotype; however retain androgen responsiveness) [13]–[14]. As shown in Figure 2(Bi–ii), all CaP cell lines RC77T/E, PCa2b and E006 exhibited a higher expression of BMI1 protein than in normal cells RC77N/E. These data (Fig. 2A–B) suggest a possibility that expression of intracellular BMI1 protein may be correlated with the secretory BMI1 levels in human tissues and may play a role in aggressiveness of human CaP.

### BMI1 is a secretory protein: Detection in serum-free media from CaP cell cultures

The presence of BMI1 in the apical region of prostate epithelial cells and stromal region prompted us to hypothesize that it could be a secretory protein in nature. To test our hypothesis we asked if BMI1 is secreted by tumor cells under culture conditions. In order to detect BMI1 protein in culture media of CaP cells, we employed Slot-blot technique. Cells at a confluency level of 80% were allowed to grow in fresh serum-free media for 24 h. As evident from Fig. 2C, serum free media (harvested from CaP cells culture) tested positive for BMI1 protein. Notably, media collected from cultures of epithelial cells representative of normal and BPH condition exhibited very low BMI1 protein (Fig. 2C).

### Quantification of secretory BMI1 in culture media of cells representing CaP in Caucasian and African-American men

By employing a human specific BMI1-ELISA technique, we were able to detect and quantify BMI1 protein secreted by cells representing CaP in Caucasian and African-American men (Fig. 2D). We determined secreted BMI1protein levels (a) in the culture media of normal, BPH1, and (b) in the culture media of tumor cells representing various cancer types. BMI1 was detected in the culture media of normal prostate cells (RWPE1; 0.45 ng/ml media) and interestingly the levels of BMI1 were not elevated in BPH1 cells (Fig. 2D). As compared to normal RWPE1 cells, CaP cells exhibited increased secretory BMI1 protein levels in media (Fig. 2D). LNCaP, C42b, PC3 and Du145 cells were observed to secrete BMI1 protein in a range of 1.3–3.4 ng/ml of media (Fig. 2D). It is noteworthy that BMI1 secreted protein was observed in the serum-free culture media of all types of CaP cell lines representing from normal RWPE1 to lesser aggressive LNCaP to castration-resistant prostate cancer (CRPC) cells C42b through highly aggressive Du145 and PC3 cells. This finding corroborates with the data obtained CaP patients representing progressive stages of disease who were analyzed for serum-BMI1 protein levels. Notably, media collected from the cultures of epithelial cells E006 (derived from African American CaP patient) exhibited significantly high BMI1 protein (Fig. 2D). On the contrary, the culture media of prostate stromal cells (WPMY1), normal colon epithelial cells (FHC) and normal pancreatic ductal epithelial cells (PDE) did not exhibit any secreted BMI1 levels (data not shown). Interestingly, secreted BMI1 levels were not to be observed in all types of pancreatic (Kras-mutant PDE, E6E7-Ras and E6E7-Ras-st) and colon carcinoma cell lines (SW480, HCT116), but only in highly aggressive pancreatic cell lines AsPC1 (at very low levels; data not shown) and colon HT29 cells (data not shown). The ELISA data of secretory BMI1 conforms to our observations in immunhistochemical analysis of CaP tissue specimens where we observed an increased stromal staining for BMI1 protein. This would be the first report showing BMI1 as a secretory protein from tumor cells.

### Secreted BMI1 in the culture media is directly related to intracellular BMI1 of tumor cells

**Figure 3:**
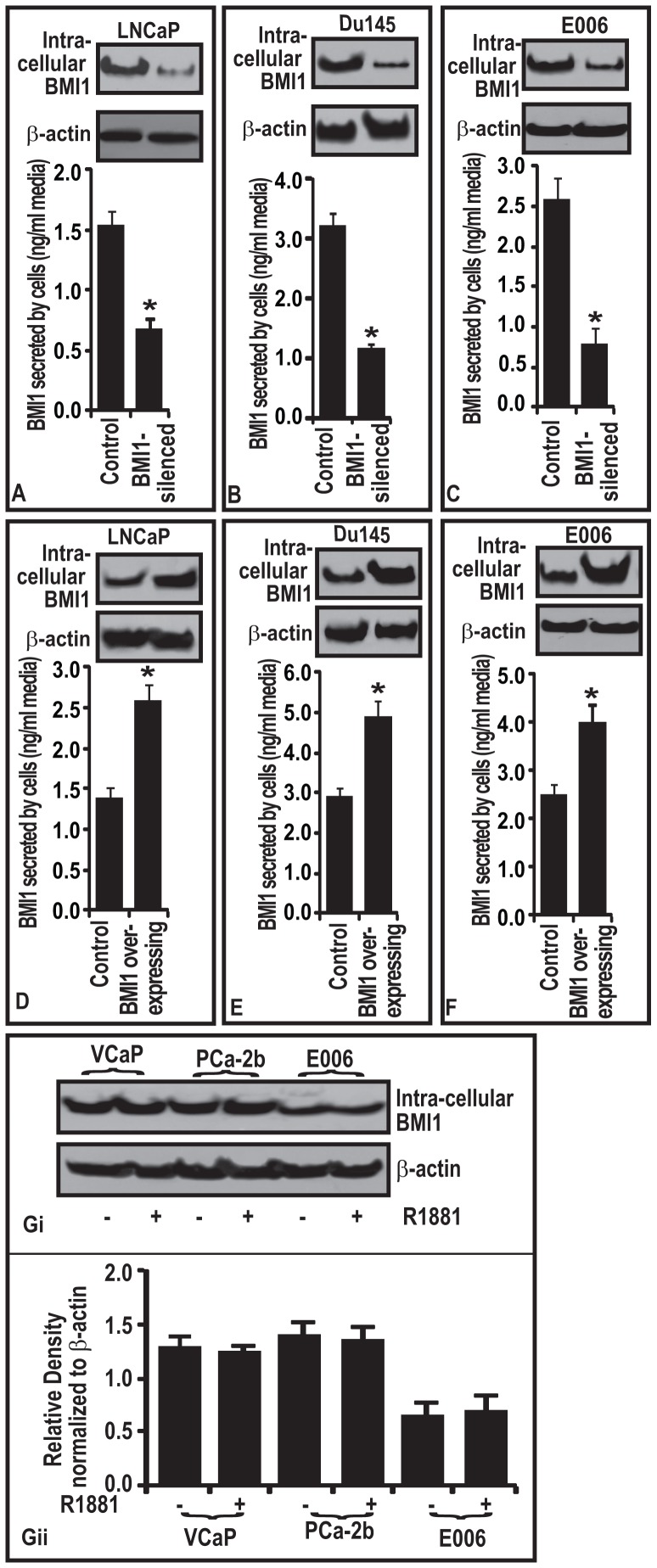
Secretory BMI1 is correlated with its intracellular levels in prostatic tumor cells and is independent of androgen. (**A–F**) Figure represents the effect of (A–**C**) BMI1-silencing and (**D–F**) BM11-overexpression on the level of secreted BMI1 protein in conditional media of different cells as assessed by ELISA assay. Equal loading of protein was confirmed by reprobing immunoblots for β-actin. Each bar in the histogram represents mean ± SE of 3 independent experiments, *represents P<0.05. (**Gi**) Figure represents the level of BMI1 protein in androgen (R1881) treated and non-treated CaP cells as assessed by immunoblot analysis. Equal loading of protein was confirmed by reprobing immunoblot for β-actin. (**Gii**) Histogram showing the densitometry analysis of immunoblots of BMI1. *, P<0.05; black bar in gray box, median values.

Since BMI1 was observed to secrete in the culture media, we sought to determine if this secretion is related to intracellular BMI1. We employed a two-way approach where BMI1 was either knocked-down or overexpressed in CaP cells. After 24 h post transfection, BMI1-suppressed and BMI1-overexpressed cells were cultured in the serum-free media for further 24 h. Next, serum free media from transfected cell cultures were harvested and analyzed for BMI protein by employing an ELISA. BMI1 protein levels were observed to be highly reduced in BMI1-knocked-down cells and increased in BMI1-overexpressed cells (Fig. 3A–F; p<0.05). These data show that BMI1-silenced tumor cells significantly secrete low levels of BMI1 protein and BMI1-overexpressed CaP cells secreted significantly high levels of BMI1 protein in the culture media, the data suggest that intracellular BMI1 is directly correlated with the secretory BMI1 protein levels (Fig. 3A–F; p<0.05). We speculate that increase in the intracellular BMI1 levels in CaP cells amounts to its subsequent release by epithelial cells into the extracellular space and causes a spike in the secretory BMI1 protein levels.

### BMI1 expression in cells representing CaP in Caucasian and African-American men is independent of influence of androgen

The differences between races in androgen concentrations and sensitivity are considered as important factors for the racial disparities in CaP [20]. However, androgen concentrations do not always correlate to PSA in cancer patients and sometimes mislead the outcome [21]. We next asked if the BMI1 levels in humans CaP disease has a correlation with presence or absence of androgen. For this purpose we selected VCaP (representing androgen-independent CRPC phenotype in Caucasian population), E006 (representing androgen-dependent non-tumorigenic prostatic epithelial cells from African-American population) and PCa-2b (androgen responsiveness CRPC cells from African-American population). Androgen treatment (R1881; 1 nM) of VCaP, E006, and PCa-2b cells did not cause significant change in the levels of BMI1 protein (Fig. 3Gi–ii; p<0.05) thus suggesting that BMI1 expression is independent of androgen status. This data is significant because aggressive CaP in both Caucasian and African-American is often Androgen independent [6], [19].

### Detection of BMI1 protein in blood of human CaP patients

**Figure 4:**
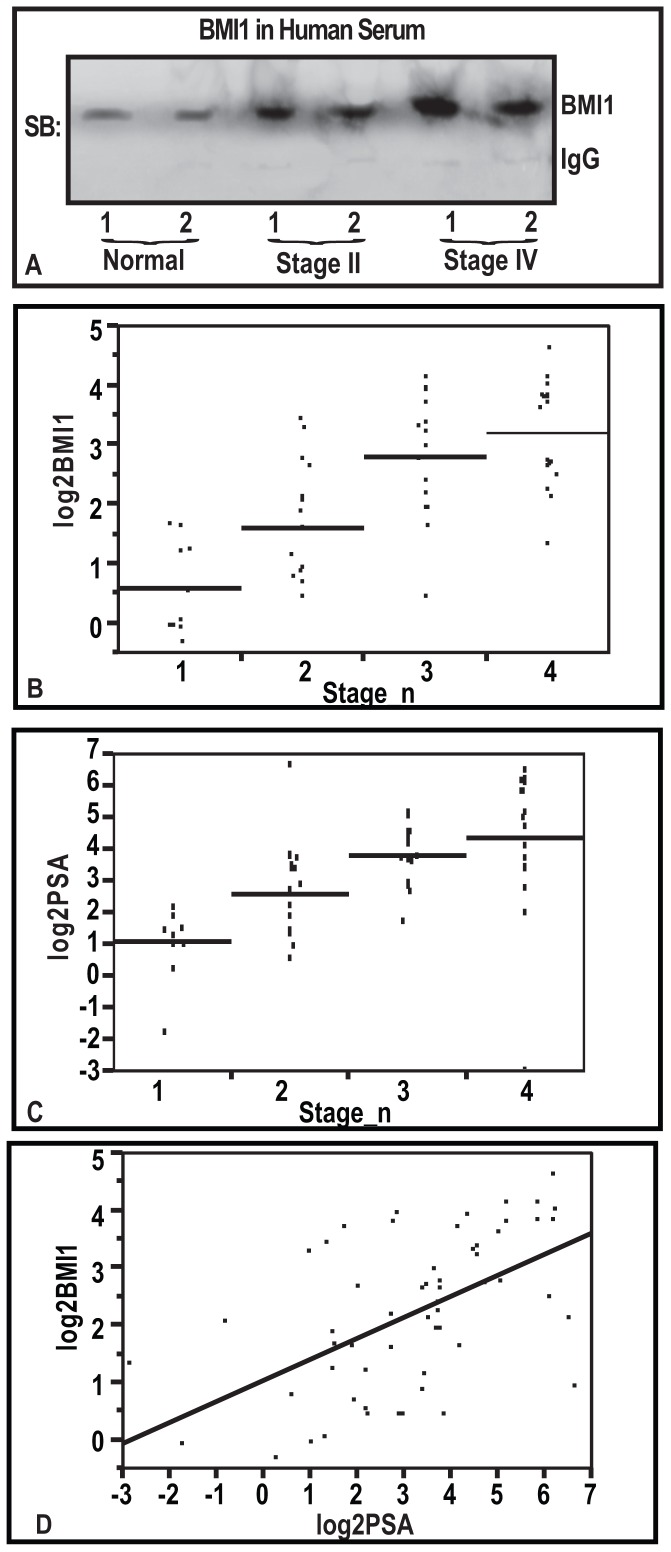
Measurement of serum-BMI1 protein levels in human CaP patients. (**A**) Figure represents the detection of BMI1 in human serum as assessed by Slot-blot analyses. The blot data shown here are representative of three samples. (**B**) Plot of BMI1 (ng/ml, log-2) versus CaP group rank (n = 58). Horizontal line is the group mean. (**C**) Plot of PSA (ng/ml, log-2) versus CaP group rank (n = 58). Horizontal line is the group mean. Each group (Fig. F & G) represented as 1 = Normal, 2 = Stage II, 3 = Stage III, and 4 = Stage IV CaP. (**D**) Figure represents the correlation between serum-PSA (ng/ml, log-2) and serum-BMI1 (ng/ml, log-2) (Spearman r = 0.58, p<0.001) in 58 men. Line is from simple linear regression.

Since, BMI1 protein was observed to be secreted by human prostatic epithelial cells in vitro. We next asked if BMI1 could be detected in the serum of CaP patients. By employing Slot-blot analysis, we determined the levels of BMI1 protein in albumin-free cleared sera, prepared from human blood (randomly selected from normal and CaP patients). As evident from the Fig. 4A, BMI1 protein was detected in the serum of CaP patients.

### Serum-BMI1 protein levels increase progressively with CaP development in human patients

We next asked if serum-BMI1 protein levels bear translational relevance as a potential biomarker for staging and development of CaP disease in humans. For this purpose we investigated if serum-BMI1 protein levels exhibit a significant difference with respect to different stages of CaP. We determined serum-BMI1 levels in a cohort of 58 human subjects representing normal disease free condition, and different CaP stages, viz., normal (n = 10), Stage II CaP (n = 16), Stage III CaP (n = 15), and Stage IV CaP (n = 17). The average serum-BMI1 protein levels in normal human subjects (n = 10) were estimated to be approximately 1.72±0.30 ng/ml of serum (Table 1). The serum BMI1 level for each patient is provided in Table 2. Serum-BMI1 levels were lower in normal human subjects than in CaP patients. BMI1 protein levels in human CaP patients was 3.91±0.60 ng/ml in stage II CaP, 8.55±1.95 ng/ml in stage III CaP and 10.84±2.44 ng/ml in stage IV CaP (Table 1). These data showed that mean serum-BMI1 protein levels were progressively increased with increasing stage of CaP disease in humans (r = 0.72, p<0.001, Fig. 4B). These data suggest that serum-BMI1 protein levels possess a translational potential to be developed as a novel serum-biomarker for CaP disease however further studies in a large cohort of patients are warranted.

**Table 1:** Serum-BMI1 protein levels in human prostate cancer patients.

		ng/ml serum		
Stage	Number of Human Subjects	PSA (mean ± SE)	BMI1 (mean ± SE)	Average GS
Normal	10	2.60±0.54	1.72±0.30	None
Stage II	16	12.82±9.67	3.91±0.60*	6.0±0.092
Stage III	15	16.77±3.91*	8.55±1.95*	6.9±0.12
Stage IV	17	38.04±12.15*	10.84±2.44*	7.8±0.40

GS represents Gleason score;

* Represents p<0.05.

**Table 2:** Comparative analysis of serum-PSA and serum-BMI1 in prostate cancer patients vis-à-vis Gleason score.

						Therapy					ng/ml serum	
S.N.	Age	Stages	GS	DA	MS	CT	RT	HT	Serum collected during	CaP Type	PSA	BMI1
1	78	-	-	-	-	-	-	-	-	-	1.18	0.81
2	57	-	-	-	-	-		-	-	-	2.8	3.2
3	66	-	-	-	-	-	-	-	-	-	4.43	2.32
4	70	-	-	-	-	-		-	-	-	3.63	3.09
5	65	-	-	-	-	-	-	-	-	-	2.01	0.97
6	80	-	-	-	-	-		-	-	-	2.02	0.97
7	78	-	-	-	-	-	-	-	-	-	0.29	0.94
8	57	-	-	-	-	-		-	-	-	2.45	1.02
9	66	-	-	-	-	-	-	-	-	-	2.72	2.37
10	70	-	-	-	-	-		-	-	-	4.51	1.46
11	73	II	3+3	71	N	N	Y	N	Remission	AC	10.38	3.83
12	73	II	3+3	51	N	N	Y	Y	Treatment	AC	11.27	4.39
13	83	II	3+3	77	N	Y	N	Y	Treatment	AC	2.51	10.96
14	74	II	3+3	73	N	N	Y	N	Treatment	AC	13.51	4.24
15	70	II	3+3	57	N	N	Y	Y	Remission	AC	1.48	3.72
16	82	II	3+3	79	N	N	Y	Y	Treatment	AC	13.39	4.83
17	72	II	3+3	71	N	N	N	Y	Remission	AC	***0.57***	***2.16***
18	72	II	3+2	51	N	N	Y	N	Treatment	AC	6.62	3.04
19	81	II	3+3	77	N	Y	Y	Y	Treatment	AC	10.56	2.24
20	57	II	3+3	57	N	N	N	N	Detection	AC	1.91	7.80
21	60	II	3+3	60	N	N	N	N	Detection	AC	4.62	2.37
22	64	II	3+3	60	N	N	N	Y	Treatment	AC	3.75	2.61
23	75	II	4+3	75	N	N	N	N	Detection	AC	2.72	1.37
24	47	II	3+3	47	N	N	N	N	Detection	AC	14.45	2.89
25	58	II	3+3	58	N	Y	N	Y	Treatment	AC	19.91	2.35
26	63	II	3+3	63	N	N	N	N	Detection	AC	25.33	3.67
27	72	III	3+4	66	N	N	Y	Y	Remission	AC	13.04	5.26
28	78	III	4+3	77	N	N	N	Y	Treatment	AC	35.87	17.52
29	67	III	3+3	62	N	N	Y	N	Remission	AC	7.02	15.49
30	73	III	3+4	67	N	N	Y	Y	Remission	AC	3.30	13.23
31	77	III	4+4	73	N	N	N	Y	Treatment	AC	20.06	15.30
32	69	III	4+3	64	N	N	Y	Y	Remission	AC	23.16	9.39
33	70	III	3+4	66	N	N	Y	Y	Remission	AC	21.83	10.01
34	73	III	3+3	77	N	N	N	Y	Treatment	AC	33.35	6.79
35	65	III	4+3	62	N	N	Y	N	Remission	AC	12.29	7.95
36	68	III	3+4	66	N	N	N	Y	Treatment	AC	6.45	4.56
37	70	III	3+4	69	N	N	N	Y	Treatment	AC	7.70	4.37
38	60	III	3+4	60	N	N	Y	N	Treatment	AC	12.72	3.86
39	54	III	3+4	54	N	N	N	N	Detection	AC	23.18	7.51
40	65	III	4+3	65	N	N	N	N	Detection	AC	13.54	3.83
41	57	III	4+3	57	N	Y	N	Y	Treatment	AC	18.08	3.13
42	69	IV	3+3	66	N	N	N	Y	Remission	AC	35.96	14.02
43	85	IV	4+4	80	N	N	N	Y	Treatment	AC	57.67	17.69
44	52	IV	9+0	51	Li, L	Y	Y	Y	Treatment	AC	32.07	12.21
45	68	IV	3+4	64	N	N	Y	Y	Remission	AC	57.64	14.29
46	80	IV	4+4	76	N	N	Y	Y	Treatment	AC	74.57	16.34
47	57	IV	6+4	53	Li	Y	N	Y	Treatment	AC	67.97	5.59
48	76	IV	3+3	65	N	Y	N	Y	Treatment	AC	89.70	4.33
49	74	IV	2+1	74	B	N	Y	Y	Treatment	AC	25.91	6.71
50	73	IV	6+2	62	B, L	N	Y	Y	Treatment	AC	17.27	13.23
51	70	IV	4+5	70	N	N	N	N	Treatment	AC	71.32	24.89
52	47	IV	4+4	46	N	Y	N	Y	Treatment	AC	6.78	14.09
53	60	IV	4+5	60	N	N	N	N	Detection	AC	***0.25***	***3.50***
54	59	IV	3+5	59	N	N	N	N	Detection	AC	11.0	6.58
55	72	IV	4+5	72	N	N	N	N	Detection	AC	13.16	4.79
56	63	IV	5+4	63	N	N	N	N	Detection	AC	4.02	5.39
57	75	IV	4+3	73	N	N	Y	N	Treatment	AC	10.35	6.30
58	75	IV	4+4	64	N	N	N	Y	Treatment	AC	71.16	14.41

N represents NO; Y represents Yes; AC represents adenocarcinoma; CaP represents prostate cancer; GS represents Gleason score; DA represents diagnosis age; MS represents metastatic site; CT represents chemotherapy; RT represents radiation therapy, HT represents hormonal therapy; L represents lung; Li represents Liver ; B represents bone; Bold and italic represent values in patients with low PSA and high BMI1 levels.

### Serum-BMI1protein levels were correlated with serum PSA levels

Next we investigated if an increase in BMI1 during CaP developments has a correlation with PSA levels in these patients (n = 58). In this Cohort of CaP patients, association of PSA with progression of CaP was also observed (r = 0.57, p<0.001, Fig. 4C). The serum PSA level for each patient is provided in Table 2. BMI1 was modestly correlated with PSA (r = 0.58, p<0.001, Fig. 4D). BMI1 remained significantly (p<0.001) when adjusting for PSA in a regression model predicting cancer stage group.

## Discussion

A prognostic biomarker provides evidence about a patient's eventual outcomes from a disease independent of a given therapy, whereas a predictive-biomarker estimates the likelihood of response/benefit to a specific therapy in a specific context [22]. PSA still remains the marker of choice for CaP diagnosis, prognosis, and active surveillance. However, PSA has several limitations [23]–[26]. For example, sipuleucel-T is known to improve survival without having an impact on early PSA levels [27]. PSA progression during CRPC therapy is reported to be prognostic for overall survival but likewise is not a surrogate for overall survival [22]. Some CaP types such as neuroendocrine tumors, produce little if any PSA and decreased secretion of PSA in patients suffering from ductal CaP has also been reported [23], [25]. In these cases, PSA alterations do not correlate well with clinical benefit [23], [25]. There is an unmet need to identify a robust and reliable biomarker which can detect disease progression in patients in whom PSA is not a reliable indicator. Thus, the development of biomarker(s) that can correlate with disease stage along the course of tumor progression is important for intervention and treatment of disease, especially chemoresistant CaP.

In the current study, we provide evidence that BMI1 secretory protein has high potential to be developed as a reliable serum-biomarker for human CaP. We provide compelling evidence that BMI1 protein is (i) secreted by tumor cells in greater amounts proportionate to tumor stage and grade, (ii) detectable in blood of human CaP patients in an order of increasing tumor/Gleason Score grade and, (iii) detectable in some CaP patients which exhibit very low levels. Further, we showed a good correlation (r = 0.58) between secretory-PSA and secretory-BMI1 in the serum of human CaP patients. Thus, expression of PSA, along with the detection of BMI1 in serum and biopsy tissue samples, may offer a new approach for CaP diagnosis, prognosis, and active surveillance. We suggest that serum-BMI1 could bring under surveillance some cases in which PSA levels do not correlated with disease progression.

African-American men exhibit the worst prognosis of CaP disease which could be due to several reasons [20], [28]–[29]. It is being suggested that absence of a reliable predictive biomarker for African American CaP is one of the contributory factors for the failure of prognosis in African-American CaP patients. Clinical studies suggested significant differences and in the levels of PSA of between Caucasian and African-American CaP patients [20]. PSA is androgen-dependent and its expression is regulated by androgen receptor [30]. The difference in androgen concentrations between African-American and Caucasian is considered as important factor for the racial disparities in CaP prognosis [20]. It has been reported that androgen receptor expression is 81% higher in African-American CaP patients that in Caucasian and high androgen receptor stimulation has been considered as one of the reasons for CaP development at a younger age with rapid progress in African-American men [29]. Changes in PSA may be informative in patients treat with anti-androgen therapy. However, changes in serum-PSA do not always predict the action of therapy or the disease condition [27], [31]–[33]. Furthermore, in neuroendocrine or small cell prostate cancer, very little or no PSA is produced, and therefore PSA changes do not correlate with disease status [33]. Thomson et al. [34] reported that CaP can be detected in approximately 15% of men with normal or low levels of total PSA level. This data questions the validity of PSA as a global serum-biomarker for men. Our study in this context is significant as we provide evidence that BMI1 could be a reliable predictive secretory biomarker for both the races especially African-American CaP. This is evident from our data we show that E006 cell (derived from African American CaP patient) does not express PSA [14], however secrete BMI1 in culture media (Fig. 2D). Notably, E006 cell line also expressed intracellular BMI1 (Fig. 2B). This data suggest that BMI1 could be used as a biomarker for even those cases in African-American men who exhibit very low PSA levels but develop CaP disease. This corroborates to our data in Caucasian men, where we were able to detect BMI1 in patients which exhibited very low PSA levels (Table 2). Furthermore, BMI was found to independent of androgen and thus, it may be very useful as a prognostic biomarker in patients with both early as well as advanced prostate cancer.

Thus, analysis of BMI1 in tissue biopsies and serum analysis may serve as a prognostic biomarker in CaP and may ultimately lead to monitoring therapeutic response during CaP treatment protocols. We suggest that BMI1 stands out as a promising molecule to be developed as an ideal serum-biomarker for prognosis of CaP in humans. We suggest that this study has high translational potential however, warrants further investigation in a big cohort of human patients. It is imperative that BMI1 as a biomarker be studied rigorously in parallel with drug development (which is underway in our laboratory), given the potential to maximize benefit and management of CaP disease in Caucasian as well as African-American patients, that in turn will minimize the harms and costs to society.

## References

[1] Siegel R, Naishadham D, Jemal A (2012) Cancer statistics. CA Cancer J Clin 62: 10–29. 22237781 10.3322/caac.20138

[2] Bickers B, Aukim-Hastie C (2009) New molecular biomarkers for the prognosis and management of prostate cancer-the post PSA era. Anticancer Res 29: 3289–3298. 19661347

[3] Levy DA, Jones JS (2011) Management of rising prostate-specific antigen after a negative biopsy. Curr Urol Rep 12: 197–202. 21344195 10.1007/s11934-011-0177-1

[4] Meulenbeld HJ, Bleuse JP, Vinci EM, Raymond E, Vitali G, et al (2012) Randomized phase II study of danusertib in patients with metastatic castration-resistant prostate cancer after docetaxel failure. BJU Int doi: 10.1111/j.1464-410X.2012.11404.x. 10.1111/j.1464-410X.2012.11404.x 22928785

[5] Casanova-Salas I, Rubio-Briones J, Fernández-Serra A, López-Guerrero JA (2012) miRNAs as biomarkers in prostate cancer. Clin Transl Oncol PMID 22855165. 10.1007/s12094-012-0877-0 22855165

[6] Madu CO, Lu Y (2010) Novel diagnostic biomarkers for prostate cancer. J Cancer 1: 150–177. 20975847 10.7150/jca.1.150 PMC2962426

[7] Fowler JE Jr, Bigler SA, Farabaugh PB (2002) Prospective study of cancer detection in black and white men with normal digital rectal examination but prostate specific antigen equal or greater than 4.0 ng/mL. Cancer 94: 1661–1671. 11920526 10.1002/cncr.10446

[8] Siddique HR, Saleem M (2012) Role of BMI1, a Stem Cell Factor in Cancer Recurrence and Chemoresistance: Preclinical and Clinical Evidences. Stem Cells 30: 372–378. 22252887 10.1002/stem.1035

[9] Kang MK, Kim RH, Kim SJ, Yip FK, Shin KH, et al (2007) Elevated BMI1 expression is associated with dysplastic cell transformation during oral carcinogenesis and is required for cancer cell replication and survival. Br J Cancer 96: 126–133. 17179983 10.1038/sj.bjc.6603529 PMC2360223

[10] Cenci T, Martini M, Montano N, D'Alessandris QG, Falchetti ML, et al (2012) Prognostic Relevance of c-Myc and BMI1 Expression in Patients With Glioblastoma. Am J Clin Pathol 138: 390–396. 22912356 10.1309/AJCPRXHNJQLO09QA

[11] Campbell PM, Groehler AL, Lee KM, Ouellette MM, Khazak V, et al (2007) K-Ras promotes growth transformation and invasion of immortalized human pancreatic cells by Raf and phosphatidylinositol 3-kinase signaling. Cancer Res 67: 2098–2106. 17332339 10.1158/0008-5472.CAN-06-3752

[12] Hayward SW, Wang Y, Cao M, Hom YK, Zhang B, et al (2001) Malignant transformation in a nontumorigenic human prostatic epithelial cell line. Cancer Res 61: 8135–8142. 11719442

[13] Theodore S, Sharp S, Zhou J, Turner T, Li H, et al (2010) Establishment and characterization of a pair of non-malignant and malignant tumor derived cell lines from an African American prostate cancer patient. Int J Oncol 37: 1477–1482. 21042716 10.3892/ijo_00000800 PMC3132581

[14] Koochekpour S, Maresh GA, Katner A, Parker-Johnson K, Lee TJ, et al (2004) Establishment and characterization of a primary androgen-responsive African-American prostate cancer cell line, E006AA. Prostate 60: 141–152. 15162380 10.1002/pros.20053

[15] Saleem M, Adhami VM, Zhong W, Longley BJ, Lin CY, et al (2006) A novel biomarker for staging human prostate adenocarcinoma: overexpression of matriptase with concomitant loss of its inhibitor, hepatocyte growth factor activator inhibitor-1. Cancer Epidemiol Biomarkers Prev 15: 217–227. 16492908 10.1158/1055-9965.EPI-05-0737

[16] Siddique HR, Liao DJ, Mishra SK, Schuster T, Wang L, et al (2012) Epicatechin-rich cocoa polyphenol inhibits Kras-activated pancreatic ductal carcinoma cell growth in vitro and in a mouse model. Int J Cancer 131: 1720–1731. 22190076 10.1002/ijc.27409

[17] Siddique HR, Mishra SK, Karnes RJ, Saleem M (2011) Lupeol, a novel androgen receptor inhibitor: implications in prostate cancer therapy. Clin Cancer Res 17: 5379–5391. 21712449 10.1158/1078-0432.CCR-11-0916 PMC4573593

[18] Glinsky GV, Berezovska O, Glinskii AB (2005) Microarray analysis identifies a death-from-cancer signature predicting therapy failure in patients with multiple types of cancer. J Clin Invest 115: 1503–1521. 15931389 10.1172/JCI23412 PMC1136989

[19] Parray A, Siddique HR, Nanda S, Konety BR, Saleem M (2012) Castration-resistant prostate cancer: potential targets and therapies. Biologics 6: 267–276. 22956858 10.2147/BTT.S23954 PMC3430091

[20] Bigler SA, Pound CR, Zhou X (2011) A retrospective study on pathologic features and racial disparities in prostate cancer. Prostate Cancer 2011: 239460. 22135747 10.1155/2011/239460 PMC3206508

[21] Asbell SO, Raimane KC, Montesano AT, Zeitzer KL, Asbell MD, et al (2000) Prostate-specific antigen and androgens in African-American and white normal subjects and prostate cancer patients. J Natl Med Assoc 92: 445–449. 11052458 PMC2608532

[22] Armstrong AJ, Eisenberger MA, Halabi S, Oudard S, Nanus DM, et al (2012) Biomarkers in the Management and Treatment of Men with Metastatic Castration-Resistant Prostate Cancer. Eur Urol 61: 549–559. 22099611 10.1016/j.eururo.2011.11.009 PMC3445625

[23] Sasaki T, Komiya A, Suzuki H, Shimbo M, Ueda T, et al (2005) Changes in chromogranin a serum levels during endocrine therapy in metastatic prostate cancer patients. Eur Urol 48: 224–9. 16005374 10.1016/j.eururo.2005.03.017

[24] Shariat SF, Semjonow A, Lilja H, Savage C, Vickers AJ, et al (2011) Tumor markers in prostate cancer I: blood-based markers. Acta Oncol 50: 61–75. 10.3109/0284186X.2010.542174 PMC3571678 21604943

[25] Spiess PE, Pettaway CA, Vakar-Lopez, Kassouf W, Wang X, et al (2007) Treatment outcomes of small cell carcinoma of the prostate: a single-center study. Cancer 110: 1729–1737. 17786954 10.1002/cncr.22971

[26] Fradet Y (2009) Biomarkers in prostate cancer diagnosis and prognosis: beyond prostate-specific antigen. Curr Opin Urol 19: 243–246. 19325493 10.1097/MOU.0b013e32832a08b5

[27] Kantoff PW, Higano CS, Shore ND, Berger ER, Small EJ, et al (2010) Sipuleucel-T immunotherapy for castration-resistant prostate cancer. N Engl J Med 363: 411–422. 20818862 10.1056/NEJMoa1001294

[28] Smith DS, Carvalhal GF, Mager DE, Bullock AD, Catalona WJ (1998) Use of lower prostate specific antigen cutoffs for prostate cancer screening in black and white men. J Urol 160: 1734–1738. 9783942

[29] Gaston KE, Kim D, Singh S, Ford OH, Mohler JL (2003) Racial differences in androgen receptor protein expression in men with clinically localized prostate cancer. J Urol 170: 990–993. 12913756 10.1097/01.ju.0000079761.56154.e5

[30] Balk SP, Ko YJ, Bubley GJ (2003) Biology of prostate-specific antigen. J Clin Oncol 21: 383–391. 12525533 10.1200/JCO.2003.02.083

[31] Armstrong AJ, Garrett-Mayer E, Ou Yang YC, et al (2007) Prostate-specific antigen and pain surrogacy analysis in metastatic hormone-refractory prostate cancer. J Clin Oncol 25: 3965–3970. 17761981 10.1200/JCO.2007.11.4769

[32] Petrylak DP, Ankerst DP, Jiang CS, Tangen CM, Hussain MH, et al (2006) Evaluation of prostate-specific antigen declines for surrogacy in patients treated on SWOG 99-16. J Natl Cancer Inst 98: 516–521. 16622120 10.1093/jnci/djj129

[33] Taplin ME, George DJ, Halabi S, Sanford B, Febbo PG, et al (2005) Prognostic significance of plasma chromogranin a levels in patients with hormone-refractory prostate cancer treated in Cancer and Leukemia Group B 9480 study. Urology 66: 386–391. 16098367 10.1016/j.urology.2005.03.040

[34] Thompson IM, Pauler DK, Goodman PJ, Tangen CM, Lucia MS, et al (2004) Prevalence of prostate cancer among men with a prostate-specific antigen level < or = 4.0 ng per milliliter. N Engl J Med 350: 2239–2246. 15163773 10.1056/NEJMoa031918

