# Correction: BMI1, Stem Cell Factor Acting as Novel Serum-biomarker for Caucasian and African-American Prostate Cancer

**DOI:** 10.1371/annotation/d612ee51-4c8e-4468-8373-3d4c503f0da5

**Published:** 2013-06-07

**Authors:** Hifzur Rahman Siddique, Aijaz Parray, Weixiong Zhong, R. Jeffery Karnes, Eric J. Bergstralh, Shahriar Koochekpour, Johng S. Rhim, Badrinath R. Konety, Mohammad Saleem

There were three errors in Column 4 of Table 2.

Column 4: Row depicting Patient number 44: The correct value should appear as 5+4.

Column 4: Row depicting Patient number 47: The correct value should appear as 5+4.

Column 4: Row depicting Patient number 50: The correct value should appear as 5+3.

